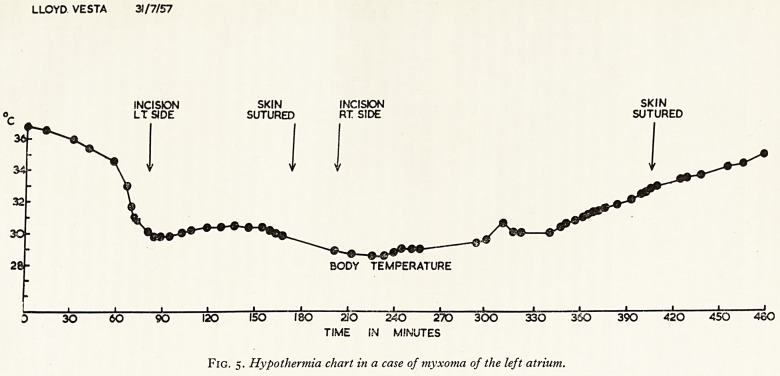# Recent Developments in Cardiac Surgery

**Published:** 1959-01

**Authors:** R. E. Horton, J. Clutton-Brock


### RECENT DEVELOPMENTS IN CARDIAC SURGERY

BY

R. E. HORTON, M.B.E., M.S., F.R.C.S.,
*Consultant Surgeon*

AND

J. CLUTTON-BROCK, F.F.A.R.C.S.,
*Lecturer in Anaesthetics*

The scope of cardiac surgery is continually changing. Operations such as those [illegible] mitral stenosis, patent ductus arteriosus and coarctation of the aorta are now w[illegible] established. These cases rarely require special investigation and have passed into t[illegible] routine list of any general surgeon who is familiar with the conduct of thoracoto[illegible] and its complications. Other operations are made possible by the use of hypotherm[illegible] and a further group remains in which successful results will be achieved after a s[illegible] extra-corporal circulation has been perfected. Up to the present, too few cases requiri[illegible] surgery under hypothermia have been referred to us for treatment; in selecting cas[illegible] for surgery it must be remembered that the presence of advanced cardiac patholo[illegible] or cardiac failure considerably adds to the mortality and now that hypothermia carri[illegible] very little risk, it is unwise to with-hold surgery until signs of heart strain appe[illegible] When the experimental work on cardiac by-pass has been completed the same princi[illegible] will apply if the work is to be carried out in patients with any hope of success.

### DIAGNOSIS

Exact preoperative diagnosis is imperative for safe cardiac surgery. In addition [illegible] routine procedures such as radiography and electrocardiography it may be necess[illegible] to perform cardiac catheterisation and cine radiography after injecting radio-opaq[illegible] dye into the right ventricle. In acquired abnormalities of the mitral valve in which t[illegible] exact functional abnormality is in doubt the left atrium may be punctured for press[illegible] recording through a bronchoscope introduced into the left bronchus. This techniq[illegible] was introduced by Allison and was subsequently developed in the United States. It [illegible] possible to thread a fine nylon tube through the needle in the left bronchus and th[illegible] tube is fed into the left ventricle and aorta. Pressure readings taking across the aort[illegible] and mitral valves give an indication of their function which is valuable in some cases.

Surgical treatment may now be classified under three main headings:

(1) Operations which can be performed under standard conditions.
(2) Operations which require hypothermia.
(3) Operations which can only be performed with cardiopulmonary by-pass or t[illegible] heart lung machine.

### OPERATIONS UNDER HYPOTHERMIA

Animal experiments in hypothermia were begun in Bristol in 1954 with a view [illegible] correcting atrial septal defects in man. At this time skin cooling was employed usi[illegible] refrigerated glycol circulating in rubber tubes packed into blankets. This method w[illegible] successfully used to make atrial septal defects in animals but the time taken to co[illegible] the animals was very great (Fig. 1). Other workers found that immersion in a cold [illegible] iced bath achieved quicker cooling. In this method the animal gets a blanket of warm[illegible] water adjacent to him which insulates him from the cold water of the bath. It w[illegible] found that circulating water prevented this insulating effect and more rapid cooling [illegible]

FIG. 1. *Cooling curves.*

· · · *Small dog cooled by original technique of circulating glycol at* 3° *C through a blanket containing rubber tubing. Atrial septal defect created, with recovery of the animal* (1954).

—— *Adult patient cooled for carotid embolectomy using immersion in running tap water and later control with plastic blanket as described in text.*

warming was possible (Fig. 2). Some further cooling takes place when the patient is taken out of the cold bath as a result of circulation of blood through cold skin. This "after cooling" can be limited to about 1°–2° C by placing the patient on a water blanket through which water is pumped at 40° C (Plate IV). By varying the temperature of the circulating water considerable control of the patients temperature is possible.

Hypothermia permits total arrest of the circulation for periods up to 10 minutes at 29° C and we have used this for a variety of cases. Temperatures below 28° C carry a risk of ventricular fibrillation. In addition we have used moderate hypothermia (31° C) in some poor risk cardiac cases such as severe mitral stenosis and tricuspid atresia.

During surgery we believe continuous electrocardiography to be essential and during the period of circulatory arrest and subsequent recovery electro-encephalography is routinely used. The electro-encephalogram gives information regarding the adequacy of the cerebral circulation and during periods of cardiac arrest it is used to assess the effectiveness of the surgeon's cardiac massage (Plate IV).

### CARDIOPULMONARY BY-PASS

The development of successful "heart-lung" machines in the U.S.A., stimulated us to begin work on this problem about 3 years ago. The problem is to extract blood from the cavae, to oxygenate this blood and to return it at a reasonable pressure and flow rate to the ascending aorta. The pump side of the problem is not great and most machines have foundered on failure of the oxygenator. Oxygenation and removal of carbon dioxide from blood does not present a difficult problem in itself but it must be done without damaging the blood, defibrinating it, or introducing microscopic sized bubbles. About three years ago a team consisting of Dr. Barratt-Boyes and Mr. Wilson, in addition to the authors began to study this problem and the full results of this work will shortly be published. Briefly we tried to use heterologous lungs, taken from an animal killed at the time of the experiment. In fact goats lungs were most commonly used as oxygenator to by-pass the heart and lungs of a calf. The calf proved a difficult experimental animal but some success was achieved and there were some survivors.

However, the technique presented many difficulties and work in the Departments of Surgery and Anaesthetics is now directed to the design of a satisfactory oxygenator. There can be little doubt that this problem will be mastered and direct surgery of the ventricles and valves of the heart will then be possible.

FIG. 2. *Experiment to show the ready control of temperature which can be obtained by changes in the ambient temperture of circulating water.*

In this experiment the animal was a fully grown unshaved goat.

### MITRAL STENOSIS

No special investigations are required in cases of mitral stenosis unless there is a combined cardiac and pulmonary disability. In such cases cardiac catheterisation may be advisable as a method of estimating left atrial pressure. Bronchoscopic catheterisation of the left atrium gives a direct left atrial pressure reading and if the diagnosis of mitral incompetence is in question pressure tracings across the mitral valve may be helpful. If the mitral valve restenoses after valvotomy it is best treated by a transventricular operation using a mechanical dilator, and in fact recent experience suggests that much better results can be achieved by the use of a dilator when the valve resists digital commissurotomy.

So far the relief of mitral incompetence has eluded surgeons but treatment may be possible when the heart-lung machine has been perfected.

### PATENT DUCTUS ARTERIOSUS

It is now nearly 20 years since the first ligation of a patent ductus arteriosus (Gross 1939, Keele and Tubbs 1940). The operation is now well established and it is generally accepted that a patent ductus should always be closed surgically. In a few cases cardiac enlargement or failure takes place in early infancy and such an event demands early intervention. The youngest case in our series was a baby of 10 weeks who was in obvious cardiac failure and a number of other babies with cardiomegaly have had the ductus closed before the age of 2 years. Most cases are diagnosed at 5 years by the physician making a routine school examination. These cases are asymptomatic but they should be treated surgically without delay. It is absolutely unjustifiable to wait in the hope that spontaneous closure will take place after the age of 5 years. Although this faint possibility exists it is far outweighed by the increasing risk of surgery in older children and adults.

Many physicians have records of occasional patients with patent ductus arteriosus who have survived to the age of 50 or more but deterioration is likely in the 3rd or 4th decades and safe surgery can only be offered in childhood. Irene Cade reported the first 50 patients treated in the Department of Surgery at Bristol in 1954; the number now exceeds 100 without mortality. Most cases are still treated by triple ligation but when the ductus is large division between Potts' clamps is preferred.

### COARCTATION OF THE AORTA

In this condition too, the main principles of surgery have been established. The natural history has been reviewed by Campbell and Baylis; the blood pressure rises steadily to an average figure of 190-105 at 17 years and there is a risk of sudden fatality during the 3rd decade. Few of those who survive to 40 years remain asymptomatic.

The case for resection of a coarctation about the age of 7 to 8 years is a strong one. At this age the operation can be completed with comparative ease and safety. The aorta is healthy and holds sutures well. Later it becomes atheromatous and after the age of 15 years the operation carries a much greater hazard though it is still well worth while. In a review of cases treated in the Department of Surgery, Bristol, and also in Manchester, Milnes Walker and Haxton (1954) noted a considerable drop in blood pressure after resection though it often took some months for the full effect to take place. There is a tendency for an aneurysm to form below the coarctation and in the older patients it may be necessary to resect a considerable length of aorta. For this reason an operation for coarctation should never be undertaken unless an arterial graft is available.

There has recently been a plea for surgical intervention in infants with coarctation and heart failure. It is doubtful if this course is the right one. Careful nursing and digitalis may pull an infant through a difficult first few months of life. Surgery at best can give a suture line which is the size of the aorta in infancy and if the scar does not stretch a considerable narrowing will develop as growth takes place. It is also im possible at present to determine that the heart failure is not due to co-existing fibro elastosis.

### PULMONARY STENOSIS

Pulmonary stenosis occurs as part of the abnormality referred to as the tetralogy o Fallot. In this section reference is made only to what is commonly designated pu pulmonary stenosis. This is in fact poor terminology, for an associated atrial septa defect is common.

Stenosis of the pulmonary valve varies in degree but always results in hypertroph of the right ventricle and a pressure gradient across the pulmonary valve. Eventuall the right ventricle shows signs of strain and failure and if an atrial septal defect i present blood will be shunted from right to left and cause cyanosis. An importa bundle of heart muscle, the crista supraventricularis, takes part in the general hype trophy of the right ventricle and this may eventually be so great that in systole th infundibulum of the right ventricle is almost completely occluded. This can be clearl shown by cine radiography. Brock (1957b) has called attention to this infundibul obstruction and describes the heart as "muscle-bound". Valvotomy at this stage ma not relieve the obstruction to the right ventricle and we have experience of two patien who died after satisfactory valvotomy from failure of the right ventricle. Kirklin (195 has called attention to the fact that a fall in right ventricular pressure may take pla gradually after valvotomy and suggests this is due to regression of myocardial hype trophy. If patients are left until secondary obstruction to the infundibulum has take place the opportunity for safe relief of pulmonary stenosis by any route has been los The crista supraventricularis could be resected through the pulmonary valve aft valvotomy but the effect of division of such an important muscle bundle may be seriou

It is our present practice to operate under hypothermia. An excellent view of t valve is obtained and complete division of the valve to the valve ring is possible, wi about 5 minutes of circulatory arrest. Figure 3 and Plate V show the hypotherm chart and incision in a patient referred by Dr. Apley. Recently we have been doing th operation through a vertical sternotomy. Access is excellent and the post operati course is smoother because both pleural cavities are intact.

### ATRIAL SEPTAL DEFECT

There are three main anatomical types of atrial septal defect (Fig 4).

(*i*) *Persistent ostium primum defect*

In this type of defect the lower edge is continuous with the atrioventricular val ring. Closure of such a defect by direct suture would result in serious interferen with function of the valves and must be avoided. This type of defect is often associat with disability and enlargement of the heart in childhood. Other signs may inclu left ventricular hypertrophy or heart block.

A more serious variety of this anomaly is the persistent atrioventricular canal which a ventricular septal defect is also present and may be continuous through t valve ring with the atrial septal defect. Some of these patients are mongols.

Treatment presents a serious problem at present. Simple suture under hypotherm is contraindicated and it will probably be necessary to insert a prosthesis of polyvi alcohol sponge with the heart isolated and circulation maintained with the heart-lu machine.

(*ii*) *Persistent ostium secundum* (*fossa-ovalis defect*)

The essential feature of this defect is a clear margin of septum between the def and the atrioventricular valve ring. Secundum defects vary somewhat in size and m be fenestrated. When the defect is small patients remain symptomless for many ye In many cases however deterioration takes place in the 3rd and 4th decades. Wh this happens the time for safe surgery has passed and Bedford, Kirklin, Swan and oth

FIG. 3. *Hypothermia chart in a patient operated on for pulmonary stenosis. Note the way in which the patient's temperature was lowered about one degree just before the period of circulatory arrest by circulating cold water through the blanket.*

FIG. 4. *Anatomical types of Atrial Septal Defect. A. Septum primum defect. B. Secundum defect. C. High defect with anomalous venous return.*

[Face page 12

*General view of operating room during hypothermia. In addition to bath and water blanket the picture shows from left to right, electrical manometer, electrocardiograph and electroencephalograph, anaesthetic apparatus and thermistor thermometer.*

PLATE V

*Bilateral thoracotomy incision used for major cardiotomy. This patient had pulmonary valve stenosis. He was treated by direct vision valvotomy under hypothermia.*

PLATE VI

*Myxoma removed from left atrium.*

FIG. 5. *Hypothermia chart in a case of myxoma of the left atrium.*

(1957) believe that all uncomplicated cases of atrial septal defect of this type should operated on before the age of 20 years. Now that hypothermia carries such slig intrinsic risk there can be little doubt as to the logic of this even though most of t patients will be symptom free at the time of surgery.

(*iii*) In the third type the defect is above the foramen ovale and lies immediat below the superior vena cava. Associated anomalous vanous return is commonly se in this group; i.e. one or more pulmonary veins drain into the right atrium.

These defects can be closed under direct vision using hypothermia but care must taken to close the defect in front of the pulmonary vein openings and it may eventua prove better to operate on this type of defect with cardiac by-pass.

### INTRA-CARDIAC TUMOURS

A variety of intracardiac tumours have been described but of these the myxoma most common and of greatest surgical importance. This is a polypoid tumour wh generally arises in the left atrium and causes obstruction to the circulation by structing the mitral valve. During periods of exercise the increased blood flow m sweep the tumour into the mitral orifice plugging it and causing total arrest of the c culation and temporary syncope. Most cases are diagnosed as mitral stenosis with a mitral murmur. Only one successful operation has so far been recorded from t country and that in a man of 25 years who had a four months history of cardiac illn (Chin and Ross 1957). The approach to a myxoma of the left atrium is through t right atrium and the atrial septum. A direct approach through the left atrium is alm certainly doomed to failure because of the entry of air into the left side of the heart a so into the coronary circulation.

In one patient operated on at the Bristol Royal Infirmary the diagnosis was stron suspected before cardiotomy. This was a woman of 55 years referred by Dr. Sut who had been diagnosed as having mitral stenosis for some years and had been pra cally bed-ridden with cardiac failure for about a year. There were no murmurs b clinical and catheter evidence of severe pulmonary hypertension. The mitral va was explored in the usual way but under hypothermia (Fig. 5). The valve was nor and the myxoma was identified arising from the atrial septum just above the mitral val The incision was closed, the position changed and a bilateral thoracotomy done. T circulation was controlled in the usual way and the right atrium opened; the at septum was incised and the tumour removed from the left atrium (Plate VI). T septum was closed and the atrium clamped. Circulatory arrest was for 10 minu The circulation was slow in becoming properly re-established but with the assista of cardiac massage a normal electroencephalogram was obtained and consciousn restored. Unfortunately she died about 18 hours after the operation.

### AORTIC STENOSIS

Considerable series of cases of both congenital and acquired aortic stenosis h been published. Marquis and Logan (1955) have called attention to the fact that congenital cases sudden death may occur without previous dyspnoea on exertion. six of their patients operated on by the transventricular route aortic incompetence w produced in four. As aortic incompetence is a more damaging lesion than ao stenosis these results must give cause for anxiety. We have operated on two patie with congenital aortic stenosis. One is greatly improved clinically and the syst pressure is 10 mm Hg higher but a faint diastolic murmur is now present, some t years after the operation. The other is too recent for assessment but has a 30 mm r in systolic blood pressure.

No case of acquired aortic stenosis has been referred for surgical treatment thou Brock (1957a) has reported results of 130 operations with a good result in 70 per c of the survivors. In assessing the indication he suggests a pressure gradient of m than 50 mm. Hg. as shown by left ventricle and brachial artery puncture. T

operative routes are available. The transventricular route carries the disadvantages that there is a high incidence of ventricular fibrillation and that the operation is blind. Serious aortic incompetence has undoubtedly been caused in some cases and Lillehei (1957) comments that most surgeons cannot hide some disappointment over the results.

In one patient who died in hospital with severe aortic stenosis while under medical treatment a post-mortem valvotomy was performed by the transventricular route. When the dilator was opened the valve was felt to give way but when it was inspected it was found that two cusps had given way but the adherent commissures had not been split. For these reasons the open transaortic view under hypothermia is favoured because the commissures could then be deliberately divided. Our second case of congenital aortic stenosis was treated this way. It is probable that the opening should be something less than a cut right out to the valve ring. There is a distinct difference in principle here from the operation on the pulmonary valve. The aortic valve has to hold a diastolic pressure some 10 times that exerted on the pulmonary valve and incompetence is much more easily produced. Indeed it is well known that in severe systemic hypertension a normal aortic valve is sometimes incompetent.

Congenital subaortic valvar stenosis is a more promising surgical problem which can be dealt with through the ascending aorta and the aortic valve. Such an obstruction can be relieved without damaging the aortic valve and without risk of incompetence. The operation can be done with hypothermia and circulatory arrest or using cardiac by-pass.

### FALLOT'S TETRALOGY AND VENTRICULAR SEPTAL DEFECT

Since the work of Blalock, Potts and Brock, patients with Follat's Tetralogy have derived considerable benefit from indirect operations (e.g. subclavian-pulmonary or aortic pulmonary anastomosis) or blind direct operations. We have had experience of each of these techniques and there are some excellent long term results. But other cases are less satisfactory and with the development of the heart-lung machine it should be possible to close a ventricular septal defect and to overcome the pulmonary stenosis. Not all cases can be suitable for such a procedure however. In some cases the pulmonary artery is absent and in others so small that closure of a V.S.D. would be followed by right ventricular failure consequent on the circulatory obstruction in the pulmonary artery. It is likely that shunt operations will have a permanent place in the more severe types of this effect.

### CONCLUSION

It has been the purpose of this article to give an account of the work which has been done in Bristol both in clinical and experimental cardiac surgery.

In 1954 when work on hypothermia began few other centres in this country had any experience of the technique and in none was the experience great. Since the technique has been standardised round about 1955 only one adult with an atrial septal defect has been referred for surgery. A considerable number of children have been seen in consultation but it has been considered that these had septum primum defects and were unsuitable for repair by this technique.

It has only been possible in such a brief review to mention our current views on a few of these problems. It has not been possible to discuss more complicated disorders such as those of total and partial anomalous venous return, transposition of the great vessels, tricuspid atresia and related defects.

Finally it should be emphasised that intracardiac surgery can only be successful if it is the work of a team. Preoperative consultation between physician, surgeon and radiologist is essential. Co-operation at the time of operation and after it is no less important. It is because of this team work that such vast progress has been made in the past decade and there can be little doubt that a properly equipped centre with an active cardiac team can still contribute much to our knowledge of cardiology and al[illegible] to the relief of human suffering.

We wish to express our thanks to the physicians and radiologists who have c[illegible] operated in the development of cardiac surgery in Bristol. In addition we should li[illegible] to thank Professor Messervy for his continued assistance with the provision of expe[illegible] mental facilities in the Surgical Department of the Veterinary School at Langfo[illegible] Without his co-operation much of this work would never have been done. Finally [illegible] thank Professor Milnes Walker who has done so much for cardiovascular surg[illegible] in this country and who has helped and encouraged us throughout.

We are grateful to Mr. Badrick for technical help in the theatre and for draw[illegible] the graphs.

### REFERENCES

1. Bedford, D. Evan, Sellors, T. Holmes, Somerville, W., Belcher, J. R., Besterman, E.M. (1957) *Lancet*, **1**, 1255.
2. Brock, R. C. (1957a) *Brit. Med. J.*, **1**, 1019.
3. Brock, R. C. (1957b) *The Anatomy of Congenital Pulmonary Stenosis*. Cassell, London.
4. Cade, Irene (1954) *Brit. J. Surg.*, **42**.
5. Campbell, M. and Baylis, J. H. (1956) **18**, 475.
6. Chin, E. F., Ross, D. N., (1957) *Brit. Med. J.* **1**, 1447.
7. Gross, R. E. (1939), *Ann. Surg.*, **110**, 321.
8. Keele, K. D. and Tubbs, O. S. (1940), *St. Bart's Hosp. J.*, **1**, 175.
9. Kirklin, J. W., Connelly, D. C., Ellis, F. H., Burchell, H. B., Edwards, J. E., Woods, E. [illegible] (1953) *Circulation*, **8**, 849.
10. Kirklin, J. W., Weidman, W. H., Burroughs, J. T., Burchell, H. B., Wood, E. H. *Circ*[illegible]*tion* (1956), **13**, 825.
11. Lillehei, C. W., Paneth, M. (1957) *Ann. Rev. Med.*, **8**, 99.
12. Marquis, R. M., Logan, A. (1955) *Brit. Heart J.*, **17**, 373.
13. Swan, H., Blount, S. G., Virtue, R. W., (1955) *Surgery*, **38**, 858.
14. Walker, R. Milnes, H., (1954) *Brit. J. Surg.* **42**.

## Figures and Tables

**Fig. 1. f1:**
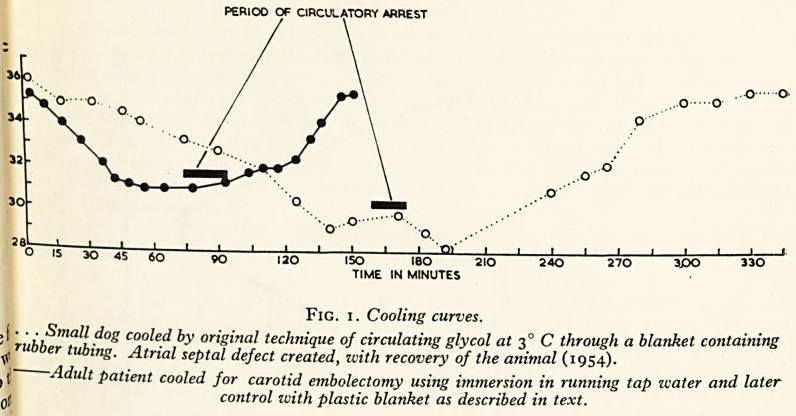


**Fig. 2. f2:**
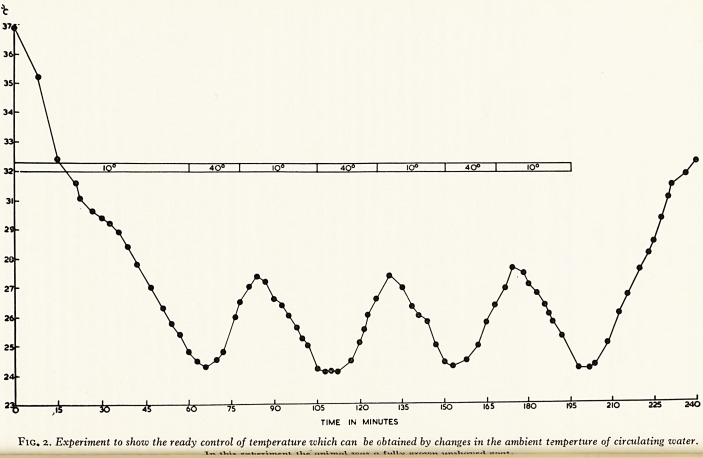


**Fig. 3. f3:**
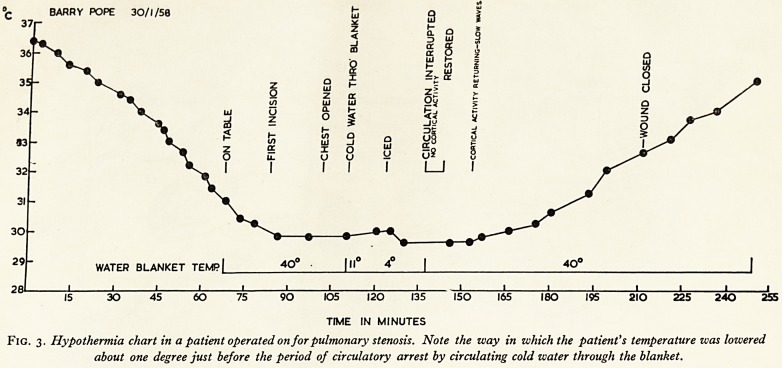


**Fig. 4. f4:**
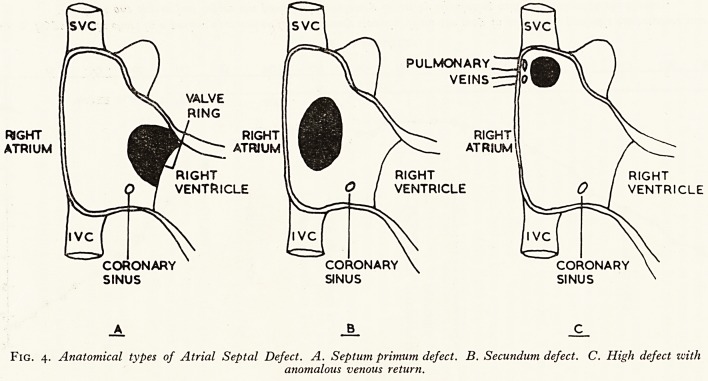


**Figure f5:**
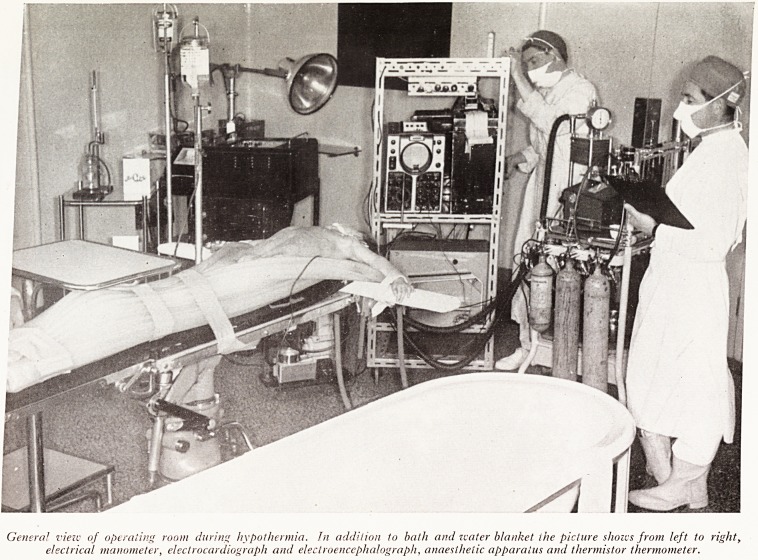


**Figure f6:**
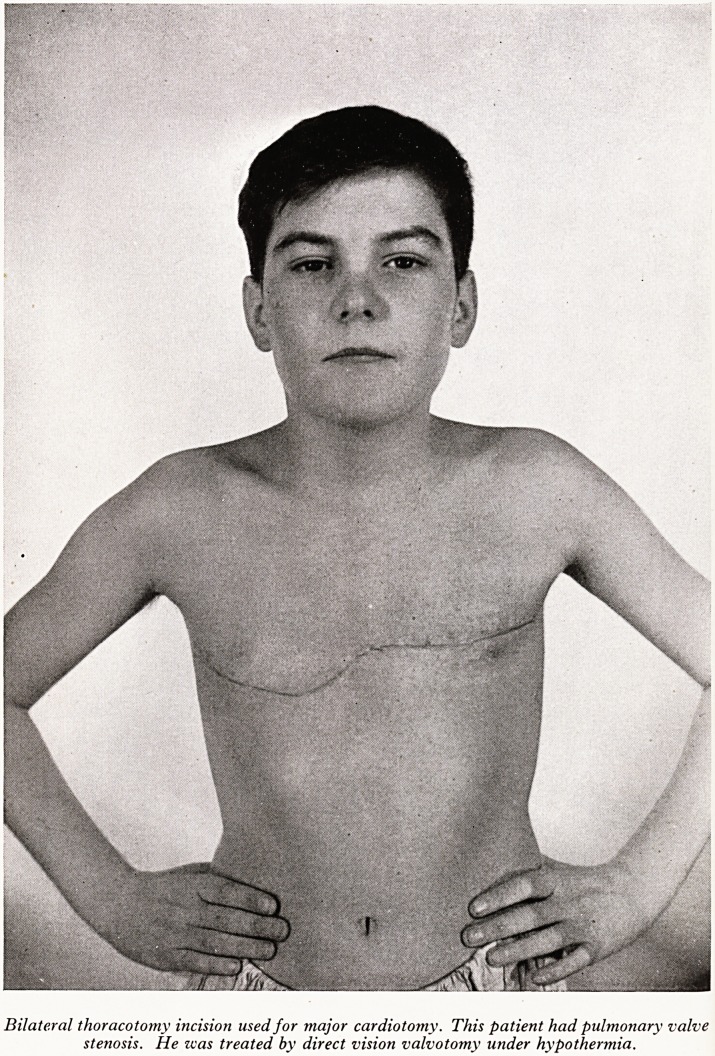


**Figure f7:**
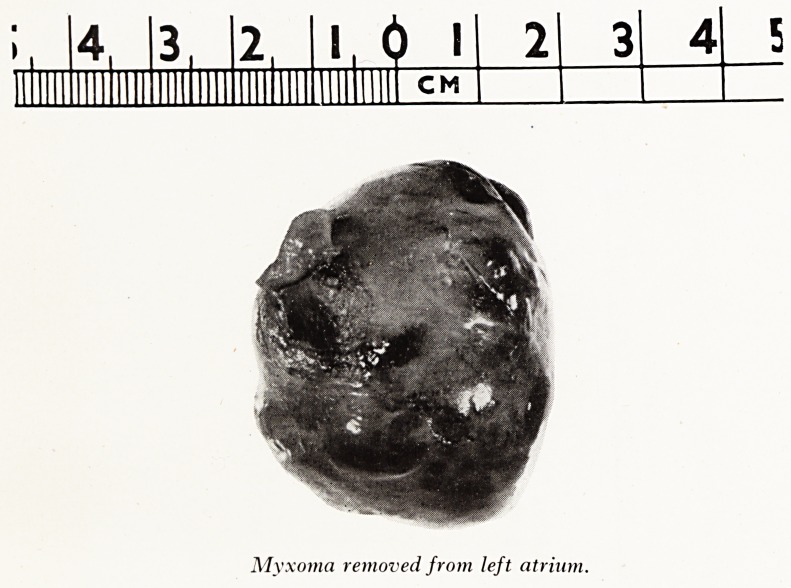


**Fig. 5. f8:**